# Personality, Psychopathology, Life Attitudes and Neuropsychological Performance among Ritual Users of Ayahuasca: A Longitudinal Study

**DOI:** 10.1371/journal.pone.0042421

**Published:** 2012-08-08

**Authors:** José Carlos Bouso, Débora González, Sabela Fondevila, Marta Cutchet, Xavier Fernández, Paulo César Ribeiro Barbosa, Miguel Ángel Alcázar-Córcoles, Wladimyr Sena Araújo, Manel J. Barbanoj, Josep Maria Fábregas, Jordi Riba

**Affiliations:** 1 Human Experimental Neuropsychopharmacology. IIB Sant Pau, Barcelona, Spain; 2 Centre d’Investigació de Medicaments. Servei de Farmacologia Clínica, Departament de Farmacologia i Terapèutica, Universitat Autònoma de Barcelona. Hospital de la Santa Creu i Sant Pau, Barcelona, Spain; 3 International Center for Ethnobotanical Education, Research and Service, ICEERS, Halsteren, Netherlands; 4 Unidad Farmacología Humana y Neurociencias, Instituto de Investigación Hospital del Mar-IMIM, Parc de Salut Mar, Universidad Autónoma de Barcelona, Barcelona, Spain; 5 Center UCM-ISCIII for Human Evolution and Behavior, Madrid, Spain; 6 Instituto de Etnopsicología Amazónica Aplicada (IDEAA), Barcelona, Spain; 7 Independent Researcher, Barcelona, Spain; 8 Departamento de Filosofia e Ciências Humanas, Universidade Estadual de Santa Cruz (UESC), Ilhéus, Bahia, Brazil; 9 Departamento de Psicología Biológica y de la Salud, Facultad de Psicología, Universidad Autónoma de Madrid (UAM). Cantoblanco, Madrid, Spain; 10 Prefeitura Municipal de Rio Branco, Conselho Estadual de Educação do Estado do Acre, Rio Branco, Acre, Brazil; 11 Centro de Investigación Biomédica en Red de Salud Mental, CIBERSAM, Barcelona, Spain; Catholic University of Sacred Heart of Rome, Italy

## Abstract

Ayahuasca is an Amazonian psychoactive plant beverage containing the serotonergic 5-HT_2A_ agonist *N*,*N*-dimethyltryptamine (DMT) and monoamine oxidase-inhibiting alkaloids (harmine, harmaline and tetrahydroharmine) that render it orally active. Ayahuasca ingestion is a central feature in several Brazilian syncretic churches that have expanded their activities to urban Brazil, Europe and North America. Members of these groups typically ingest ayahuasca at least twice per month. Prior research has shown that acute ayahuasca increases blood flow in prefrontal and temporal brain regions and that it elicits intense modifications in thought processes, perception and emotion. However, regular ayahuasca use does not seem to induce the pattern of addiction-related problems that characterize drugs of abuse. To study the impact of repeated ayahuasca use on general psychological well-being, mental health and cognition, here we assessed personality, psychopathology, life attitudes and neuropsychological performance in regular ayahuasca users (n = 127) and controls (n = 115) at baseline and 1 year later. Controls were actively participating in non-ayahuasca religions. Users showed higher Reward Dependence and Self-Transcendence and lower Harm Avoidance and Self-Directedness. They scored significantly lower on all psychopathology measures, showed better performance on the Stroop test, the Wisconsin Card Sorting Test and the Letter-Number Sequencing task from the WAIS-III, and better scores on the Frontal Systems Behavior Scale. Analysis of life attitudes showed higher scores on the Spiritual Orientation Inventory, the Purpose in Life Test and the Psychosocial Well-Being test. Despite the lower number of participants available at follow-up, overall differences with controls were maintained one year later. In conclusion, we found no evidence of psychological maladjustment, mental health deterioration or cognitive impairment in the ayahuasca-using group.

## Introduction

Ayahuasca is a psychotropic tea obtained from *Banisteripsis caapi* and *Psychotria viridis,* two plant species native to the Amazon Basin [1]. Ayahuasca has traditionally played a central role in Amazonian shamanism and in more recent times it has become the sacrament of various syncretic religious groups that have exported its use and increased its popularity worldwide [2]. Attesting this expansion, the anthropological and ethnographical bibliography on its modern religious use includes more than 400 scientific papers, book chapters, PhD theses and articles in popular magazines written in ten different languages. Some estimate there are around 20,000 regular religious ayahuasca users in the twenty-three countries where the so called “ayahuasca religions” are present [3]. In the US, Canada, Holland, and Brazil, federal laws protect the religious use of ayahuasca and in Peru it has recently been declared part of the National Cultural Heritage [4].

Despite the legal protection enjoyed in some countries, ayahuasca use is not without controversy. *P. viridis* contains the hallucinogen *N,N*-dimethyltryptamine (DMT; [5]), a compound listed in the 1971 Convention on Psychotropic Substances. However, no plants (natural materials) containing DMT are at present controlled under the said convention [6]. Analogously to other serotonergic hallucinogens, DMT is thought to elicit its psychotropic effect through stimulation of the 5-HT_2A_ receptors [7], [8]. However, unlike other hallucinogens DMT is not active when administered orally, as it is readily metabolized to 3-indoleacetic acid by monoamine oxidase [9]. However, *B. caapi* contains harmala alkaloids that reversibly block the metabolic breakdown of DMT, leading to psychoactivity [10], [11].

Clinical studies have shown that ayahuasca induces a modified state of awareness that includes dream-like imagery with eyes closed, increased insight and intense emotions [10], [12]. CNS effects can also evidenced as time-dependent increases in the relative energy of the beta band of the electroencephalogram [11], [13], [14]. Effects start between 30 and 45 minutes, peak between 90 and 120 minutes and are resolved by 240 minutes [10], [12]. Physiological modifications include moderate increases in blood pressure, elevations of blood cortisol and prolactine and lymphocyte redistribution [10], [11], [12], [14]. Pharmacodynamic changes closely follow the plasma concentrations of DMT, which peaks at 90–120 min and shows an elimination half-life of one hour [10].

The increasing number of individuals using ayahuasca on a regular basis has raised public health concerns [2]. Drugs of abuse such as heroin, cocaine, alcohol or amphetamines share a common neurobiological mechanism which involves the so-called “neural reward system”, inducing functional changes in brain structures related to pleasure such as the striatum and the dopaminergic ventral-tegmental area [15]. The activation of this neural circuit is considered to play a crucial role in modulating the consequences of drug abuse, which may include psychological, medical, legal, employment and family problems [16]. DMT, on the other hand is a serotonergic drug, binding to postsynaptic 5-HT_2A_ receptors [17], [18]. Although some studies have found that hallucinogenic drugs like psilocybin or LSD (lysergic acid diethylamide) may also modulate dopaminergic neurotransmission [19]–[21], a neuroimaging study using SPECT (Single Photon Emission Computerized Tomography) found that ayahuasca increases regional brain blood flow in frontal and paralimbic areas [22], but did not find any changes in reward-related regions such as the striatum or the midbrain. Nevertheless, in a prior study by our group [23], we assessed addiction severity in two samples of long-term members of the ayahuasca religions and we did not find participants to be “addicted” to the tea. Nor did we observe the deleterious psychosocial effects commonly associated with drugs of abuse.

Despite the above findings, the question remains as to whether the continued use of ayahuasca may have an impact on personality and general mental health (negative effects other than those directly related to addiction) and cognition. On the phenomenological level, the acute cognitive, emotional and perceptual modifications brought about by ayahuasca are quite intense [11] and have been described to potentially cause psychiatric complications in some individuals [24], [25]. From a biological perspective, ayahuasca induces activation of prefrontal and temporal regions of the brain [22], [26], an activatory effect probably mediated by glutamate release [7], [8], [27]. Mental health and cognitive performance of long-term ayahuasca users has not been well-studied, and the few papers published to date on the impact of chronic ayahuasca use have failed to detect negative neuropsychological [28], [29] or psychopathological [30], [31] effects. For a review see Bouso and Riba [32].

In this paper we report the results of a study specifically designed to evaluate personality, life attitudes, mental health and neuropsychological performance in a relatively large number of ritual ayahuasca users and their matched controls. The investigation involved one initial assessment and a follow-up one year later.

## Methods

### 1. Participants

Participants belonging to ayahuasca-using groups were recruited after a meeting between the research team and members of several Brazilian ayahuasca churches. The inclusion criterion was to have been taking ayahuasca for a minimum of 15 years with a frequency of at least twice a month. Control subjects were recruited to match the age, sex and educational level of ayahuasca users. Controls were only accepted if they had taken ayahuasca a maximum of 5 times. Care was taken to make sure that the majority of individuals in the comparison groups were also actively practicing some form of religion but without the ayahuasca-using component. Participants were distributed as follows:

#### 1.1. Jungle sample

Ayahuasca group: 56 ayahuasca users from a community within the Amazon rain forest.

This group was recruited from Céu do Mapiá, a community of religious ayahuasca users in the Brazilian State of Amazonas. Céu do Mapiá is the headquarters of the Centro Eclético da Fluente Luz Universal Raimundo Irineu Serra (CEFLURIS), an important ayahuasca church within the Santo Daime movement, with branches throughout South-America, the US, Canada, Europe and Japan. Céu do Mapià was established in 1983 by the founder of CEFLURIS, Sebastião Mota de Melo (known as Padrinho Sebastião), and it has since received migrants from other parts of Brazil, South-America, and overseas. Its current population is estimated at 600 and it includes men and women of all ages and children. CEFLURIS is a church of the Santo Daime, a syncretic religion that combines Christian, indigenous, Afro-American and esoteric traditions. The experiences attained by followers during rituals are interpreted as contact with ‘God’ and the ‘spirits’ and other archetypical ‘entities’ from their religious beliefs and doctrine (for a comprehensive study of the Santo Daime religion see MacRae [33]). The mean frequency of ritual attendance in this group was about six times per month. The estimated average lifetime exposure to ayahuasca in this group ranged between 360 and 1080 times.

#### Control group: Céu do mapiá comparison group

A group of 56 controls was recruited from Boca do Acre, the nearest town to the Céu do Mapiá community. Boca do Acre is located deep in the heart of the Amazon rain forest, has a strong agricultural economy, and is a typical small Amazonian town of about 7,000 inhabitants. Of the 56 controls, only 7 had ever ingested ayahuasca. Five participants had taken it once and the other two had taken it two times.

#### 1.2. Urban sample

Ayahuasca group: Urban-based ayahuasca users.

This group consisted of 71 members of another ayahuasca religious group called *Barquinha*, located in the city of Rio Branco. The city of Rio Branco, the capital of the State of Acre, has about 150,000 inhabitants and it hosts other branches of the Santo Daime and other ayahuasca churches such as the União do Vegetal and the Alto Santo. The frequency with which Barquinha members attended rituals in our sample was about eight times per month. The estimated average lifetime exposure to ayahuasca in this group ranged between 360 and 1440 times.

#### Control group: Urban-based comparison group

Fifty-nine controls were recruited in the city of Rio Branco as a comparison group. Of the 59, only 4 had ever ingested ayahuasca and they had all taken it once.

The study was conducted in accordance with the Declarations of Helsinki, as amended in Edinburgh 2000, and subsequent updates. All subjects signed an informed consent form prior to participation. The study was approved by the human research committee of UNINORTE University (Rio Branco, Acre State, Brazil).

### 2. Study Variables

#### 2.1. Sociodemographic variables

Age (years), sex (male/female) and years of education were used to match study and control groups. Additional sociodemographic indicators such as employment status (according to Hollingshead’s categories [23]), race, marital status and religion were recorded for comparison purposes.

#### 2.2. Personality traits: Temperament and character inventory – TCI

The TCI is based on the psychobiological model of personality developed by Cloninger and coworkers [34]. The temperament dimensions are assumed to be independently inheritable and to manifest in early development, while the character dimensions are assumed to be more influenced by sociocultural learning and maturation. The TCI has 240 items with a true/false option response. The four primary dimensions of temperament and their facets are: Harm Avoidance (HA): HA1-Anticipatory Worry vs. Uninhibited Optimism; HA2-Fear of Uncertainty vs. Confidence; HA3-Shyness with Strangers vs. Gregariousness; HA4-Fatigability and Asthenia vs. Vigor; Novelty Seeking (NS): NS1-Exploratory Excitability vs. Stoic Rigidity; NS2-Impulsiveness vs. Reflection; NS3-Extravagance vs. Reserve; NS4-Disorderliness vs. Regimentation; Reward Dependence (RD): RD1-Sentimentality vs. Insensitivity; RD3-Attachment vs. Detachment; RD4-Dependence vs. Independence; and Persistence (P). The three dimensions of character are: Self-Directedness (SD): SD1-Responsibility vs. Blaming; SD2-Purposefulness vs. Lack of Goal-Direction; SD3-Resourcefulness; SD4-Self-Acceptance vs. Self-Striving; SD5-Congrugent Second Nature; Cooperativeness (C): C1-Social Acceptance vs. Social Intolerance; C2-Empathy vs. Social Disinterest; C3-Helpfulness vs. Unhelpfulness; C4-Compassion vs. Revengefulness; C5-Integrated Conscience; and Self-Transcendence (ST): ST1-Self-Forgetfulness vs. Self-Conscious Experience; ST2-Transpersonal Identification vs. Self-Isolation; ST3-Spiritual Acceptance vs. Rational Materialism. In this study we used the Brazilian Portuguese version of the TCI adapted by Fuentes and coworkers [35].

#### 2.3. Psychopathological status: The Symptom Check-List-90-Revised – SCL-90-R

The SCL-90-R [36] is a self-report questionnaire that assesses 9 psychopathological symptomatic dimensions including 90 likert-type items that are scored from 0 to 4: Somatization (SOM), Obsessive-Compulsive (O–C), Interpersonal Sensitivity (I–S), Depression (DEP), Anxiety (ANX), Hostility (HOS), Phobic Anxiety (PHOB), Paranoid Ideation (PAR), and Psychoticism (PSY). The scale also provides 3 additional psychopathological indices: General Severity Index (GSI), Positive Symptoms Distress Index (PSDI), and Positive Symptoms Total (PST). For all the scales higher scores imply worse symptomatology. In this study we used the Brazilian Portuguese version adapted by Tosello [37].

#### 2.4. Neuropsychological performance and behavior. *The Stroop Color and Word Test*

The Stroop test [38] assesses conflict monitoring and resolution (resistance to interference), cognitive tasks involving the anterior cingulate-dorsolateral prefrontal system and the rostroventral prefrontal cortex [39]. In this test subjects must first read a list of color names (“red”, “green”, “blue”) written in black ink. When this is completed, a list of “X” printed in different colors (red, green, blue) is presented and the subject must indicate the color in which each element is printed. Finally, a third list is presented to the participant containing a list of color names (“red”, “green”, “blue”) but printed this time in an incongruent ink color. As with the second list, the participant is asked to indicate the color in which each element is printed. The numbers of correctly read (first list) and correctly reported items (lists two and three) in 45 seconds are recorded. Dependent variables are the total number of words read (W), the total number of correctly identified colors in the second list (C) and the total number of color incongruent words read (IW). Finally, a “Resistance to Interference” measure (RI) is calculated according to the following formula: RI = IW-(CxW/C+W). Better performance is reflected as higher scores on IW and RI.

### The Wisconsin Card Sorting Test (WCST)

The WCST [40] is considered a measure of executive function in that it requires strategic planning, organized searching, the ability to use environmental feedback to shift cognitive set, goal-oriented behavior, and the ability to modulate impulsive responding [41]. Anatomically, the WCST involves the dorso-and ventrolateral prefrontal cortices [42]. The test consists of 4 stimulus cards placed in front of the subject. The first has a printed red triangle, the second two green stars, the third three yellow crosses, and the fourth four blue circles. Subjects are then given two decks each containing 64 response cards, which have designs similar to those on the stimulus card, varying in color, geometric form, and number. Subjects are told to match each of the cards and are given feedback as to whether they are doing right or wrong. The sorting rule is changed at fixed intervals but no warning is provided that the sorting rule has changed. There is no time limit to perform the test. The following dependent variables were assessed: Number of Total Errors, Number of Perseverative Errors, Number of Non-Perseverative Errors, Number of Achieved Categories and Failures to Maintain Set.

### The Letter-NumberSequencing (LNS) from the WAIS-III

The LNS [43] is a measure of working memory, a task involving dorso-, ventrolateral and orbitofrontal prefrontal cortices [44]. Subjects are verbally presented with a random series of numbers and letters which they have to report back in a specified order, i.e, numbers in ascending order and letters in alphabetical order. Series of increasing length are presented to the subject until an error is committed. The score is the maximum number of elements in the series correctly reported by the participant. Higher scores indicate better performance.

### The Frontal Systems Behaviour Scales (FrSBe)

The FrSBe [45] is a rating scale designed to measure behaviors associated with damage to the frontal lobes and systems of the brain. This questionnaire was used to assess hypothetical frontal lobe alterations that could potentially go undetected with classical neuropsychological tests but that could have an impact on everyday life. The questionnaire comprises 46 likert-type items with 5 response options. The items are distributed into 3 subscales: Apathy/Akinesia (14 items), Disinhibition/Emotional Dysregulation (15 items), and Executive Dysfunction (17 items). Higher scores reflect worse frontal function. A global score is computed adding up the scores of the individual scales. We used the self-report version of the scale adapted to Brazilian Portuguese by our team.

### 2.5. Life Attitudes and Psychosocial Well-Being

#### The spiritual orientation inventory (SOI)

The SOI [46] is a measure of spirituality based on the humanistic model and is designed to assess the spirituality of those affiliated with traditional religion. It is a 85-Likert-type item self-report questionnaire. Items are distributed into nine major components: Transcendent Dimension, Meaning and Purpose in Life, Mission in Life, Sacredness of Life, Material Values, Altruism, Idealism, Awareness of the Tragic, and Fruits of Spirituality. Each item has 7 response options The questionnaire was adapted into Brazilian Portuguese by our team.

#### The purpose in life test (PLT)

The PLT [47] is a measure of a subject’s perceived “meaning of life” versus “existential vacuum” and is based on Victor Frankl’s Logotherapy. It consists of 20 items, each rated on a 7-point scale ranging from 1 (low purpose) to 7 (high purpose). The total score can range from 20 (low purpose) to 140 (high purpose). The questionnaire was adapted into Brazilian Portuguese by our team.

#### The psychosocial well-Being (BIEPS-Bienestar Psicosocial)

The BIEPS [48] is a measure of psychosocial well-being composed of a global and four specific dimensions: Self-Acceptance, Autonomy, Psychosocial Bonds, and Projects. It consists of 13 items with three response options (agree, nor agree nor disagree, disagree). The questionnaire was adapted into Brazilian Portuguese by our team.

### 3. Statistical Analysis

#### 3.1. Sociodemographic variables

In order to match the samples, age and years of education were compared using independent samples Student’s t test. Although not a matching variable, employment status was also compared between users and controls by means of Student’s t test. The distribution of gender, race, marital status and religion between ayahuasca users and controls in each sample were analyzed by means of χ2.

#### 3.2. Personality, psychopathology, neuropsychology and life attitude variables

Due to the longitudinal nature of the design, we were unable to contact all the participants at the second assessment. Also, due to the field nature of the study, data from some tests was lost for some subjects. In order to maximize sample size and statistical power, we used the data from all subjects available for a given test.

Individual and group scores were obtained for the different variables. For each variable a two-way analysis of variance (ANOVA) was performed with two between-subjects factors, i.e., *Group* (ayahuasca users vs. controls) and *Sample* (jungle vs. urban). Each ANOVA was performed in the first assessment and in the second assessment 8–12 months later. Results were considered significant for p values <0.05.

## Results

### 1. Sociodemographic Variables

Results concerning the sociodemographic characteristics of the samples are presented in Table 1.

**Table 1:** Sociodemographic data as means (standard deviation) for age, years of education, employment and income and as frequencies for race, marital status and religion.

	First Assessment				Second Assessment			
	Jungle Sample		Urban Sample		Jungle Sample		Urban Sample	
	Ayahuasca	Controls	Ayahuasca	Controls	Ayahuasca	Controls	Ayahuasca	Controls
**Matching variables**								
N (men/women)	56 (29/27)	56 (24/32)	71 (33/38)	59 (31/28)	39 (19/20)	49 (19/30)	39 (21/18)	19 (7/12)
Age	36 (13.46)	33.71 (12.53)	37.32 (12.77)	38.15 (12.22)	39.21 (12.90)	34.69 (12.25)	38.82 (13.06)	40.63 (11.63)
Years Education	10.55 (3.45)	10.96 (4.35)	10.27 (3.90)	11.08 (3.30)	11.08 (3.19)	11.51 (4.40)	10.87 (4.16)	12.53 (3.03)
**Additional sociodemographic variables**								
Employment	6.04 (1.68)	4.91 (2.58)**	5.80 (2.63)	5.73 (2.61)	5.79 (1.61)	5.08 (2.70)	5.82 (2.59)	5.32 (2.43)
Income	329.46 (414.06)	555.61 (1013.85)	738.11 (943.86)	1028.93 (1072.83)	519.74 (627.52)	642.96 (647.71)	713.95 (1001.25)	1065.95 (939.92)
Race								
White	40 (71.42%)	11 (19.64%)†††	38 (53.52%)	34 (57.63%)	30 (76.92%)	10 (20.41%)†††	23 (58.98%)	11 (57.89%)
Mestizos	15 (26.78%)	45 (80.36)	31 (43.66%)	21 (35.59%)	9 (23.07%)	39 (79.59%)	15 (38.46%)	6 (31.59%)
Asian	1 (1.78%)	–	1 (1.41%)	1 (1.69%)	–	–	–	1 (5.26%)
Black	–	–	1 (1.41%)	3 (5.08%)	–	–	1 (2.56%)	1 (5.26%)
Marital status								
Married	13 (23.21%)	33 (58.93%)††	25 (35.21%)	17 (28.82%)	14 (35.90%)	31 (63.26%)†	23 (58.97%)	8 (42.1%)
Remarried	1 (1.79%)	1 (1.79%)	2 (2.82%)	1 (1.69%)	–	1 (2.05%)	1 (2.56%)	–
Separated	7 (12.5%)	2 (3.57%)	10 (14.08%)	9 (15.25%)	7 (17.94%)	5 (10.20%)	4 (10.26%)	5 (26.32%)
Divorced	4 (7.14%)	–	6 (8.45%)	5 (8.47%)	4 (10.26%)	–	–	1 (5.26%)
Never Married	31 (55.36%)	20 (35.71%)	28 (39.44%)	27 (45.77%)	14 (35.90%)	12 (24.49%)	11 (28.21%)	5 (26.32%)
Religion								
Daime/Barquinha	56 (100%)	–†††	71 (100%)	–†††	39 (100%)	–†††	39 (100%)	–†††
Catholics	–	35 (62.5%)	–	30 (58%)	–	33 (67.35%)	–	12 (63.16%)
Protestants	–	15 (26.78%)	–	17 (28.81%)	–	10 (20.41%)	–	7 (36.84%)
Others	–	3 (5.36%)	–	2 (3.39%)	–	3 (61.12%)	–	–
None	–	3 (5.36%)	–	10 (16.95%)	–	3 (6.12%)	–	–

* = *p*<0.05;

** = *p*<0.01;

*** = *p*<0.001 in the Student’s t test.

† = *p*<0.05;

†† = *p*<0.01;

††† = *p*<0.001 in the χ2 test (comparison includes multiple categories).

Asterisks and crosses indicate *p* values for between group (ayahuasca vs. controls) Student’s t tests (age, education, employment and income) and χ2 tests (gender, race, marital status and religion) at baseline and at follow up for the Jungle and Urban samples. Aya. =  Ayahuasca-using group; Comp. =  comparison group.

The Jungle sample consisted in the first assessment of 56 regular ayahuasca users and 56 controls. No significant differences were found between ayahuasca users and controls in sex, age, years of education or income either in the first or second assessment. In the Jungle sample, a statistical difference was noted in employment, with the comparison group being more qualified according to the Hollingshead categories [23]. A total of 88 volunteers from the Jungle sample were assessed in a follow-up one year later: 39 from the ayahuasca group and 49 from the comparison group. No statistical differences were found. Other demographic data such as race, marital status, and religion are also shown in the table. Both ayahuasca users and controls were mainly of whites and mestizos. The predominant marital status was “never married” in the ayahuasca users, and “married” in the controls. All ayahuasca users in the Jungle sample were members of CEFLURIS, and all but 3 individuals from the control group were followers of other Christian religions (Catholicism followed by Protestantism).

The Urban sample consisted in the first assessment of 71 ayahuasca users and 59 controls. In the second assessment 58 volunteers (39 ayahuasca users and 19 comparisons) were evaluated. We found no significant differences between the groups for sex, age, years of education, income, employment status or income variables, either in the first or second assessment. Participants in both groups were mainly whites and mestizos. Regarding marital status, most volunteers in both groups were either “never married” or “married”. All ayahuasca users in the Urban sample were members of Barquinha. Most comparison subjects defined themselves as followers of traditional Christian religions (Catholicism followed by Protestantism).

### 2. Personality Traits: Temperament and Character Inventory – TCI

Mean (SD) scores on the different TCI subscales for each sample, group, and time point are shown in Table 2.

**Table 2:** TCI scales and subscales means (standard deviation).

	First Assessment					Second Assessment				
	ANOVA	Jungle Sample		Urban Sample		ANOVA	Jungle Sample		Urban Sample	
TCI subscale	df(1,223)	Ayahuasca*n* = 54	Controls*n* = 54	Ayahuasca*n* = 64	Controls*n* = 55	df(1,116)	Ayahuasca*n* = 36	Controls*n* = 39	Ayahuasca*n* = 30	Controls*n* = 15
**HA**	F = 17.73; p<0.001	14.69 (5.03)	18.30 (4.99)	15.41 (5.36)	17.53 (5.04)	F = 4.81; p = 0.030	13.78 (5.87)	17.26 (5.35)	14.63 (6.32)	16.07 (4.95)
*HA1*	F = 12.28; p<0.001	3.46 (2.13)	4.83 (2.03)	3.92 (1.93)	4.85 (2.25)	F = 5.93; p = 0.016	3.17 (1.98)	4.67 (1.92)	3.83 (2.32)	4.27 (1.75)
*HA2*	F = 8.25; p = 0.069	4.44 (1.65)	4.87 (1.82)	4.73 (1.48)	5.07 (1.31)	F = 4.29; p = 0.041	4.36 (1.74)	5.10 (1.74)	4.60 (1.65)	5.20 (1.08)
*HA3*	F = 9.28; p = 0.003	3.48 (1.99)	4.59 (1.59)	3.83 (1.78)	4.18 (1.84)	F = 5.02; p = 0.027	3.44 (2.23)	4.51 (1.68)	3.30 (1.88)	3.93 (1.91)
*HA4*	F = 6.23; p = 0.013	3.30 (1.73)	4.07 (1.97)	2.86 (2.18)	3.42 (2.10)	F = 0.01; p = 0.912	2.81 (1.85)	2.95 (1.94)	2.90 (2.32)	2.67 (2.44)
**NS**	F = 2.28; p = 0.130	17.94 (4.67)	18.04 (4.74)	16.75 (4.82)	18.62 (5.26)	F = 1.08; p = 0.300	17.58 (4.83)	17.79 (4.83)	17.30 (4.55)	15.13 (5.08)
*NS1*	F = 1.34; p = 0.248	6.50 (1.80)	6.06 (1.64)	6.02 (1.86)	5.91 (1.83)	F = 2.11; p = 0.149	6.58 (1.70)	6.13 (1.70)	6.63 (2.40)	6.00 (1.81)
*NS2*	F = 1.13; p = 0.289	3.35 (2.05)	3.39 (1.78)	3.31 (1.99)	3.84 (2.11)	F = 0.84; p = 0.360	3.11 (2.04)	3.15 (1.97)	3.23 (2.14)	2.47 (1.77)
*NS3*	F = 1.13; p = 0.288	4.65 (1.89)	4.81 (2.35)	4.48 (2.09)	5.25 (2.18)	F = 0.12; p = 0.729	4.53 (1.89)	4.95 (2.35)	4.53 (1.79)	4.40 (2.53)
*NS4*	F = 4.50; p = 0.035	3.46 (1.63)	3.78 (1.68)	2.94 (1.77)	3.62 (1.95)	F = 0.26; p = 0.609	3.36 (1.86)	3.62 (2.13)	2.90 (1.73)	2.27 (1.49)
**RD**	F = 6.98; p = 0.009	16.11 (4.15)	14.04 (2.90)	14.39 (3.33)	14.00 (3.55)	F = 0.37; p = 0.547	15.56 (3.97)	14.36 (3.23)	14.67 (3.14)	15.07 (2.37)
*RD1*	F = 0.12; p = 0.729	6.63 (1.93)	6.85 (1.64)	6.22 (1.61)	5.84 (1.76)	F = 0.98; p = 0.325	6.56 (1.70)	6.74 (1.87)	6.23 (1.67)	6.73 (1.91)
*RD3*	F = 7.68; p = 0.006	5.37 (2.09)	4.26 (1.84)	3.61 (1.54)	3.44 (1.46)	F = 1.89; p = 0.172	5.36 (1.82)	4.36 (1.99)	3.83 (1.70)	3.87 (1.30)
*RD4*	F = 9.52; p = 0.002	3.96 (1.26)	2.93 (1.40)	5.16 (1.70)	4.95 (1.64)	F = 0.38; p = 0.537	4.11 (1.19)	3.18 (2.94)	5.03 (1.73)	5.47 (1.36)
**P**	F = 2.21; p = 0.139	5.39 (1.83)	4.56 (1.71)	4.63 (1.83)	4.73 (2.00)	F = 0.13; p = 0.721	4.83 (2.08)	4.85 (1.68)	4.73 (1.74)	4.47 (1.55)
**SD**	F = 9.56; p = 0.002	21.94 (6.09)	25.76 (6.10)	20.91 (4.99)	22.09 (7.11)	F = 11.87; p = 0.001	20.0 (5.83)	23.85 (7.35)	20.00 (4.50)	24.60 (7.13)
*SD1*	F = 8.36; p = 0.004	2.91 (1.76)	3.69 (1.63)	3.80 (1.44)	3.58 (1.83)	F = 24.75; p<0.001	2.64 (1.40)	3.67 (1.46)	2.57 (1.35)	4.27 (1.71)
*SD2*	F = 4.19; p = 0.042	4.00 (1.35)	4.41 (1.39)	3.80 (1.20)	4.09 (1.22)	F = 2.83; p = 0.095	3.58 (1.13)	3.97 (1.51)	3.77 (1.00)	4.20 (1.21)
*SD3*	F = 12.32; p = 0.001	2.24 (1.24)	3.17 (1.38)	2.13 (1.09)	2.38 (1.35)	F = 3.34; p = 0.070	2.03 (1.21)	2.61 (1.62)	2.00 (1.29)	2.27 (1.28)
*SD4*	F = 12.67; p<0.001	5.57 (2.19)	7.15 (2.34)	5.11 (2.11)	5.64 (2.25)	F = 12.23; p = 0.001	5.42 (2.35)	7.13 (2.28)	4.97 (2.34)	6.40 (2.10)
*SD5*	F = 0.012; p = 0.913	7.11 (2.13)	7.39 (1.92)	6.80 (1.89)	6.58 (2.59)	F = 2.70; p = 0.103	6.39 (1.82)	7.33 (3.88)	6.70 (1.50)	7.47 (2.59)
**C**	F = 0.43; p = 0.515	25.65 (2.40)	26.09 (2.75)	25.31 (3.01)	24.31 (4.38)	F = 0.005; p = 0.947	25.11 (2.43)	26.23 (4.49)	25.30 (2.67)	24.27 (2.19)
*C1*	F = 1.71; p = 0.192	5.65 (6.89)	5.02 (1.20)	5.11 (0.81)	4.53 (1.12)	F = 0.053; p = 0.818	4.64 (0.93)	4.82 (1.07)	4.77 (0.73)	4.67 (0.61)
*C2*	F = 1.27; p = 0.261	5.02 (1.11)	4.80 (1.15)	4.73 (1.25)	4.58 (1.45)	F = 0.002; p = 0.962	5.22 (1.44)	5.08 (0.98)	5.03 (1.10)	5.20 (0.76)
*C3*	F = 7.60; p = 0.006	5.57 (0.84)	5.26 (1.25)	5.42 (0.90)	4.98 (1.10)	F = 4.20; p = 0.043	5.53 (0.91)	5.46 (0.85)	5.60 (1.07)	4.93 (0.70)
*C4*	F = 4.99; p = 0.026	4.74 (1.28)	5.20 (1.45)	4.58 (1.26)	4.98 (1.81)	F = 1.12; p = 0.292	4.50 (0.94)	5.36 (1.29)	4.47 (0.86)	4.07 (1.39)
*C5*	F = 0.08; p = 0.773	5.59 (0.96)	5.91 (1.10)	5.47 (0.91)	5.24 (1.30)	F = 3.49; p = 0.064	5.19 (0.99)	6.44 (2.51)	5.43 (1.01)	5.40 (0.99)
**ST**	F = 25.91; p<0.001	22.80 (5.25)	19.28 (6.27)	21.02 (5.72)	16.64 (6.09)	F = 7.3; p = 0.008	22.72 (5.85)	18.26 (6.99)	20.40 (4.76)	18.53 (5.60)
*ST1*	F = 4.48; p = 0.035	7.17 (2.29)	6.65 (2.39)	6.28 (2.39)	5.47 (2.27)	F = 0.51; p = 0.475	6.69 (2.65)	6.62 (3.66)	5.77 (2.24)	6.67 (2.59)
*ST2*	F = 24.10; p<0.001	7.09 (1.71)	5.63 (2.38)	6.16 (2.36)	4.73 (2.30)	F = 7.56; p = 0.007	7.08 (2.12)	5.72 (2.37)	6.43 (1.74)	5.47 (2.47)
*ST3*	F = 30.01; p<0.001	8.54 (2.21)	7.35 (2.15)	8.58 (2.04)	6.44 (2.71)	F = 17.94; p<0.001	8.94 (1.91)	7.08 (2.46)	8.20 (1.99)	6.40 (2.59)

The ANOVA column shows results for the main effect of *Group* (ayahuasca users vs. controls). df =  degrees of freedom. Ayahuasca  =  ayahuasca-using group. HA = Harm Avoidance; HA1-Anticipatory Worry vs. Uninhibited Optimism; HA2-Fear of Uncertainty vs. Confidence; HA3-Shyness with Strangers vs. Gregariousness; HA4-Fatigability and Asthenia vs. Vigor; NS = Novelty Seeking; NS1-Exploratory Excitability vs. Stoic Rigidity; NS2-Impulsiveness vs. Reflection; NS3-Extravagance vs. Reserve; NS4-Disorderliness vs. Regimentation; RD = Reward Dependence; RD1-Sentimentality vs. Insensitivity; RD3-Attachment vs. Detachment; RD4-Dependence vs. Independence; P = Persistence; SD = Self-directedness; SD1-Responsibility vs. Blaming; SD2-Purposefulness vs. Lack of Goal-Direction; SD3-Resourcefulness; SD4-Self-Acceptance vs. Self-Striving; SD5-Congruent Second Nature; C = Cooperativeness; C1-Social Acceptance vs. Social Intolerance; C2-Empathy vs. Social Disinterest; C3-Helpfulness vs. Unhelpfulness; C4-Compassion vs. Revengefulness; C5-Integrated Conscience; ST = Self-Trascendence; ST1-Self-Forgetfulness vs. Self-Conscious Experience; ST2-Transpersonal Identification vs. Self-Isolation; ST3-Spiritual Acceptance vs. Rational Materialism.

In the first assessment, the two-way ANOVA on temperament dimensions showed a main effect of *Group* (ayahuasca users vs. controls) for Harm Avoidance [F(1,223) = 17.73; p<0.001], with lower values for ayahuasca users than controls and for Reward Dependence [F(1,223) = 6.98; p = 0.009], with higher values for ayahuasca users. Despite lower mean values for Novelty Seeking, the overall comparison was not significant. No significant main effect was found either for Persistence and a trend to significance was found for the interaction between *Group* and *Sample* [F(1,223) = 3.62; p = 0.059].

The effect on Harm Avoidance was mainly driven by significantly lower scores on Anticipatory Worry [F(1,223) = 12.28; p<0.001], Shyness [F(1,223) = 9.28; p = 0.003] and Fatigability and Asthenia [F(1,223) = 6.23; p = 0.013]. This latter subscale also showed a significant effect of *Sample* [F(1,223) = 4.17; p = 0.042], with values higher in the Jungle sample.

Despite the non-significant differences found in the Novelty Seeking dimension, analysis of the different facets comprising the scale found significantly lower scores on Disorderliness for the ayahuasca-using individuals [F(1,223) = 4.50; p = 0.035].

The effect on Reward Dependence was driven by significantly higher scores for ayahuasca users in Attachment [F(1,223) = 7.68; p = 0.006] and Dependence [F(1,223) = 9.52; p = 0.002]. Significant *Sample* by *Group* interactions were found for Attachment [F(1,223) = 4.10; p = 0.044] and Dependence [F(1,223) = 4.17; p = 0.042], with higher differences between ayahuasca users and controls in the Jungle sample.

The analysis of first assessment scores on TCI character dimensions showed significantly lower scores for ayahuasca users in Self-Directedness [F(1,223) = 9.56; p = 0.002], no differences in Cooperativeness and significantly higher scores in Self-Transcendence.

The significant effect on Self-Directedness was driven by lower scores in Responsibility [F(1,223) = 8.36; p = 0.004]; Purposefulness [F(1,223) = 4.19; p = 0.042], Resourcefulness [F(1,223) = 12.32; p = 0.001], and Self-Acceptance [F(1,223) = 12.67; p<0.001]. No *Group* effects were found for Congruent Second Nature. The analysis of *Group* by *Sample* interactions indicated that effects in the Jungle sample were significantly larger for Resourcefulness [F(1,223) = 3.94; p<0.05] and showed a trend for Self-Acceptance [F(1,223) = 3.15; p = 0.077].

Despite no overall effect on Cooperativeness, the detailed analysis of the facets comprising this dimension showed a significant *Group* effect for Helpfulness [F(1,223) = 7.60; p = 0.006] and Compassion [F(1,223) = 4.99; p = 0.026]. Compared to controls, ayahuasca users showed higher and lower scores on these two scales, respectively.

Self-Transcendence scores were significantly higher in ayahuasca users than in controls [F(1,223) = 25.91; p<0.001]. All three facets assessed in this character dimension were found to be significantly higher, specifically Self-Forgetfulness [F(1,223) = 4.48; p = 0.035], Transpersonal Identification [F(1,223) = 24.10; p<0.001], and Spiritual Acceptance [F(1,223) = 30.01; p<0.001].

In the second assessment, lower scores for Harm Avoidance were again observed [F(1,116) = 4.81; p = 0.030] but not for Reward Dependence. Again no differences were found for Novelty Seeking and Persistence. The effect on Harm Avoidance was mainly driven by significantly lower scores on Anticipatory Worry [F(1,116) = 5.93; p = 0.016], Fear of Uncertainly [F(1,116) = 4.29; p = 0.041], an effect not observed in the first assessment, and Shyness [F(1,116) = 5.02; p = 0.027].

Character dimensions again showed lower scores for ayahuasca users on Self-Directedness [F(1,116) = 11.87; p = 0.001], no differences on Cooperativeness and significantly higher scores on Self-Transcendence [F(1,116) = 7.3; p = 0.008].

Lower Self-Directedness was due to lower scores on Responsibility [F(1,116) = 24.75; p<0.001] and Self-Acceptance [F(1,116) = 12.23; p = 0.001]. Again, despite no overall effect on Cooperativeness, Helpfulness remained higher in the ayahuasca-using group [F(1,116) = 4.20; p = 0.043] but no differences were seen in Compassion.

The maintained higher scores on Self-Transcendence could be attributed to Transpersonal Identification [F(1,116) = 7.56; p = 0.007] and Spiritual Acceptance [F(1,116) = 17.94; p<0.001], but not to Self-Forgetfulness.

### 3. Psychopathological Status: The Symptom Check-List-90-Revised – SCL-90-R

Mean (SD) scores on the 9 SCL-90-R dimensions for each sample, group, and time point are shown in Table 3.

**Table 3:** SCL-90-R subescales means (standard deviation).

	First Assessment					Second Assessment				
	ANOVA	Jungle Sample		Urban Sample		ANOVA	Jungle Sample		Urban Sample	
SCL-90-R	df(1,221)	Ayahuasca*n* = 54	Controls*n* = 55	Ayahuasca*n* = 63	Controls*n* = 53	df(1,119)	Ayahuasca*n* = 32	Controls*n* = 46	Ayahuasca*n* = 30	Controls*n* = 15
GSI	F = 23.59; p<0.001	0.61 (0.63)	1.06 (0.59)	0.64 (0.57)	0.96 (0.61)	F = 7.28; p = 0.008	0.49 (0.50)	0.95 (0.85)	0.59 (0.45)	0.81 (0.60)
PSDI	F = 0.124; p = 0.726	1.54 (0.52)	1.80 (0.54)	2.43 (7.26)	1.80 (0.56)	F = 0.95; p = 0.333	1.40 (0.48)	2.47 (5.82)	1.52 (0.43)	1.81 (0.82)
PST	F = 29.84; p<0.001	31.17 (22.90)	50.33 (20.04)	32.46 (22.46)	45.08 (21.44)	F = 8.36; p = 0.005	27.94 (19.91)	42.98 (22.01)	32.33 (19.45)	40.93 (22.26)
SOM	F = 7.00; p = 0.009	0.74 (0.88)	0.99 (0.76)	0.56 (0.65)	0.86 (0.76)	F = 2.13; p = 0.147	0.63 (0.73)	0.85 (0.74)	0.53 (0.62)	0.72 (0.64)
O-C	F = 19.76; p<0.001	0.83 (0.73)	1.26 (0.75)	0.82 (0.69)	1.27 (0.77)	F = 9.40; p = 0.003	0.68 (0.59)	1.13 (0.76)	0.81 (0.59)	1.20 (0.88)
I–S	F = 16.76; p<0.001	0.80 (0.71)	1.20 (0.72)	0.73 (0.59)	1.10 (0.81)	F = 12.45; p = 0.001	0.52(0.52)	1.17 (0.78)	0.64 (0.43)	0.91 (0.90)
DEP	F = 28.14; p<0.001	0.58 (0.62)	1.13 (0.70)	0.65 (0.63)	1.04 (0.70)	F = 2.81; p = 0.096	0.53 (0.58)	0.88 (0.65)	0.75 (0.66)	0.80 (0.60)
ANX	F = 18.72; p<0.001	0.43 (0.71)	0.89 (0.60)	0.48 (0.61)	0.78 (0.71)	F = 9.27; p = 0.003	0.31 (0.46)	0.63 (0.59)	0.37 (0.52)	0.70 (0.54)
HOS	F = 7.42; p = 0.007	0.55 (0.65)	0.85 (0.73)	0.65 (0.84)	0.91 (0.77)	F = 4.29; p = 0.040	0.32 (0.38)	0.68 (0.79)	0.46 (0.62)	0.61 (0.57)
PHOB	F = 20.23; p<0.001	0.37 (0.58)	0.80 (0.63)	0.37 (0.52)	0.65 (0.66)	F = 14.11; p<0.001	0.22 (0.36)	0.58 (0.57)	0.26 (0.49)	0.65 (0.57)
PAR	F = 10.95; p = 0.001	0.78 (0.80)	1.21 (0.75)	0.86 (0.74)	1.11 (0.79)	F = 7.35; p = 0.008	0.53 (0.64)	1.04 (0.73)	0.71 (0.51)	0.92 (0.89)
PSY	F = 10.09; p = 0.002	0.48 (0.69)	0.89 (0.71)	0.62 (0.61)	0.79 (0.63)	F = 4.21; p = 0.042	0.44 (0.65)	0.78 (0.65)	0.52 (0.45)	0.68 (0.77)

The ANOVA column shows results for the main effect of *Group* (ayahuasca users vs. controls). df =  degrees of freedom. Ayahuasca  =  ayahuasca-using group. GSI-General Severity Index; PSDI-Positive Symptoms Distress Index; PST-Positive Symptoms Total; SOM-Somatization; O-C-Obsessive-Compulsive; I-S-Interpersonal sensitivity; DEP-Depression, ANX-Anxiety, HOS-Hostility, PHOB-Phobic anxiety; PAR-Paranoid ideation; PSY-Psychoticism.

In the first assessment, ayahuasca users showed significantly lower scores on all 9 psychopathological dimensions, as reflected by a significant *Group* effect on Somatization [F(1,221) = 7.00; p = 0.009], Obsessive-Compulsive [F(1,221) = 19.76; p<0.001], Interpersonal Sensitivity [F(1,221) = 16.76; p<0.001], Depression [F(1,221) = 28.14; p<0.001], Anxiety [F(1,221) = 18.72; p<0.001], Hostility [F(1,221) = 7.42; p = 0.007], Phobic Anxiety [F(1,221) = 20.23; p<0.001], Paranoid Ideation [F(1,221) = 10.95; p = 0.001], and Psychoticism [F(1,221) = 10.09; p = 0.002].

The analysis of the additional indices also showed lower scores on the General Severity (GSI) [F(1,221) = 23.59; p<0.001] and Positive symptoms (PST) indices [F(1,221) = 29.84; p<0.001] and no differences with controls regarding the Positive symptoms distress index (PSDI).

In the second assessment, lower scores in the ayahuasca-using group were again observed relative to the control subjects for 7 of the 9 dimensions, i.e., Obsessive-Compulsive [F(1,119) = 9.40; p = 0.003], Interpersonal Sensitivity [F(1,119) = 12.45; p = 0.001], Anxiety [F(1,119) = 9.27; p = 0.003], Hostility [F(1,119) = 4.29; p = 0.040], Phobic Anxiety [F(1,119) = 14.11; p<0.001], Paranoid Ideation [F(1,119) = 7.35; p = 0.008], and Psychoticism [F(1,119) = 4.21; p = 0.042]. Mean scores on Somatization and Depression were lower for users than controls but the statistical analysis did not show a significant *Group* effect. Results for the additional indices replicated findings in the first assessment, with significantly lower scores for users on the GSI [F(1,119) = 7.28; p = 0.008] and the PST [F(1,119) = 8.36; p = 0.005] and no differences in the PSDI.

### 4. Neuropsychological Performance and Behavior

Mean (SD) scores on neuropsychological tests for each sample, group, and time point are shown in Table 4.

**Table 4:** Stroop, Letter-Number Sequency Task, WSCT and FrsBe means (standard deviation).

	First Assessment					Second Assessment				
	ANOVA	Jungle Sample		Urban Sample		ANOVA	Jungle Sample		Urban Sample	
Stroop	df(1,235)	Ayahuasca*n* = 56	Controls*n* = 56	Ayahuasca*n* = 71	Controls*n* = 56	df(1,136)	Ayahuasca*n* = 39	Controls*n* = 48	Ayahuasca*n* = 34	Controls*n* = 19
W	F = 21.00; p<0.001	86.36 (17.95)	77.38 (19,49)	94.11 (16.78)	82.27 (15.62)	F = 8.48 p = 0.004	91.92 (17.58)	81.65 (19.73)	86.74 (17.75)	78.00 (16.11)
C	F = 29.38; p<0.001	62.20 (12.08)	57.09 (12.58)	69.27 (15.25)	55.09 (14.11)	F = 2.88; p = 0.092	64.82 (12.58)	61.71 (9.91)	63.76 (13.39)	59.84 (9.46)
IW	F = 31.15; p<0.001	44.36 (18.81)	34.25 (8.68)	45.87 (13.78)	36.02 (11.70)	F = 3.72; p = 0.056	42.23 (8.94)	37.21 (9.48)	39.06 (9.70)	37.95 (4.35)
RI	F = 11.84; p = 0.001	8.47 (16.99)	1.83 (7.88)	5.74 (9.48)	3.14 (6.75)	F = 0.001; p = 0.974	4.37 (6.27)	2.30 (8.80)	2.57 (5.82)	4.72 (6.95)
**WSCT**	**df(1,238)**	***n*** ** = 56**	***n*** ** = 56**	***n*** ** = 71**	***n*** ** = 59**	**df(1,134)**	***n*** ** = 37**	***n*** ** = 47**	***n*** ** = 35**	***n*** ** = 19**
*N_tot*	F = 41.44; p<0.001	39.52 (21.42)	54. 13 (19.80)	33.94 (16.16)	51.42 (20.12)	F = 5.05; p = 0.026	22.97 (15.04)	36.72 (20.56)	37.65 (27.13)	41.16 (21.53)
*N_pers*	F = 39.74; p<0.001	21.02 (13.37)	30.98 (17.71)	18.13 (9.17)	31.59 (16.75)	F = 3.62; p = 0.059	12.35 (8.61)	18.26 (10.63)	18.77 (14.50)	20.68 (11.66)
*N_nonpers*	F = 12.40; p = 0.001	17.93 (11.47)	22.86 (10.61)	15.92 (9.06)	20.36 (10.23)	F = 4.43; p = 0.037	11.00 (7.72)	18.43 (12.26)	19.11 (13.69)	20.47 (11.98)
*N_cat*	F = 0.093; p = 0.761	4.61 (1.58)	3.63 (1.69)	5.25 (1.13)	5.59 (16.31)	F = 1.52; p = 0.220	5.38 (1.88)	4.45 (1.90)	7.51 (21.09)	3.68 (2.19)
*Fail*	F = 4.39; p = 0.037	1.05 (1.20)	1.11 (1.00)	0.80 (1.10)	1.39 (1.40)	F = 0.084; p = 0.773	1.30 (1.24)	1.57 (1.63)	1.57 (1.77)	1.68 (1.63)
**LNS**	**df(1,237)**	***n*** ** = 56**	***n*** ** = 56**	***n*** ** = 71**	***n*** ** = 58**	**df(1.132)**	***n*** ** = 36**	***n*** ** = 49**	***n*** ** = 32**	***n*** ** = 19**
Score	F = 21.27; p<0.001	12.66 (3.95)	11.66 (3.73)	15.68 (3.36)	12.47 (3.06)***	F = 5.52; p = 0.020	11.64 (3.37)	10.29 (3.31)	9.91 (3.47)	8.42 (3.01)
**FrSBe**	**df(1,216)**	***n*** ** = 53**	***n*** ** = 55**	***n*** ** = 59**	***n*** ** = 53**	**df(1,118)**	***n*** ** = 34**	***n*** ** = 47**	***n*** ** = 25**	***n*** ** = 16**
*Apathy*	F = 23.79; p<0.001	32.28 (5.96)	37.53 (6.64)	28.71 (6.67)	32.15 (7.03)	F = 10.62; p = 0.001	27.85 (5.75)	31.70 (7.98)	24.48 (7.42)	30.06 (8.58)
*Dishinibition*	F = 32.84; p<0.001	31.19 (6.89)	37.13 (6.80)	26.93 (6.89)	32.38 (8.73)	F = 4.05; p = 0.046	26.03 (7.70)	32.40 (8.67)	25.16 (7.17)	27.31 (9.33)
*Executive dysfunction*	F = 11.20; p = 0.001	39.25 (8.80)	41.60 (7.94)	35.53 (7.85)	40.89 (9.56)	F = 7.04; p = 0.009	34.85 (7.15)	40.23 (9.64)	36.00 (15.05)	40.13 (15.67)
*Total*	F = 31.81; p<0.001	101.68 (17.07)	116.44 (18.55)	91.17 (17.96)	105.34 (22.16)	F = 7.64; p = 0.007	88.74 (18.02)	104.83 (21.92)	82.12 (20.81)	89.63 (29.34)

The ANOVA column shows results for the main effect of *Group* (ayahuasca users vs. controls). df =  degrees of freedom. Ayahuasca = ayahuasca-using group. W-Words; C-Colors; IW-Color incongruent words; RI-Resistence to Interference. LNS = Letter-Number sequency; *N_tot*  =  number of total errors; *N_pers* = number of perseverative errors; *N_nonpers* = number of non-perseverative errors; *N_cat* = number of achieved categores; *Fail* = failures no maintain set.

### 4.1. The Stroop Color and Word Test

In the first assessment, ayahuasca-using subjects obtained higher scores on total words [F(1,235) = 21.00; p<0.001], total colors [F(1,235) = 29.38; p<0.001], number of correctly read incongruent words [F(1,235) = 31.15; p<0.001] and resistance to interference [F(1,235) = 11.84; p = 0.001].

In the second assessment, differences were only observed for total words [F(1,136) = 8.48; p = 0.004]. A trend was observed for the number of correctly read incongruent words [F(1,136) = 3.72; p = 0.056] but not for total colors or resistance to interference.

### 4.2. The Wisconsin Card Sorting Test (WCST)

The statistical analysis of the WCST in the first assessment showed a significantly lower number of Total Errors [F(1,238) = 41.44; p<0.001], Perseverative Errors [F(1,238) = 39.74; p<0.001], Non-Perseverative Errors [F(1,238) = 12.40; p = 0.001] and Failures to Maintain Set [F(1,238) = 4.39; p = 0.037] for the ayahuasca-using subjects. No differences were found in the number of achieved categories.

In the second assessment, the number of total errors was again significantly lower [F(1,134) = 5.05; p = 0.026], as were the number of Non-Perseverative Errors [F(1,134) = 4.43; p = 0.037]. Mean number of Perseverative Errors was also lower but only showed a trend to significance in the analysis [F(1,134) = 3.62; p = 0.059]. No differences were observed in Failures to Maintain Set or in the Number of Achieved Categories.

### 4.3. The Letter-NumberSsequencing (LNS) from the WAIS-III

The first assessment showed that ayahuasca users scored significantly higher on this task than their controls [F(1,237) = 21.27; p<0.001]. This difference was larger in the Urban sample, as reflected in the *Group* by *Sample* interaction F(1,241) = 5.86; p = 0.016].

One year later, in the second assessment, this overall effect was observed [F(1,132) = 5.52; p = 0.020], but the interaction was not.

### 4.4. The Frontal Systems Behaviour Scales (FrSBe)

In the first assessment, ayahuasca users showed lower values on the total FrsBe score [F(1,216) = 31.81; p<0.001], on the Apathy/Akinesia scale [F(1,216) = 23.79; p<0.001] scale, on the Disinhibition/Emotional Dysregulation scale [F(1,216) = 32.84; p<0.001], and on the Executive Dysfunction scale [F(1,216) = 11.20; p = 0.001].

The same pattern of results was obtained in the second assessment. Again lower values were obtained for the ayahuasca-using group, with main *Group* effects on the total FrsBe score [F(1,118) = 7.64; p = 0.007], on Apathy/Akinesia [F(1,118) = 10.62; p = 0.001], Disinhibition/Emotional Dysregulation [F(1,118) = 7.04; p = 0.009] and Executive Dysfunction [F(1,118) = 4.05; p = 0.046].

### 5. Subjective Life Attitudes

Mean (SD) scores on the different subscales of the 3 life attitudes tests for each sample, group and time point are shown in Table 5.

**Table 5:** SOI, PLT, and BIEPS means (standard deviation).

	First Assessment					Second Assessment				
	ANOVA	Jungle Sample		Urban Sample		ANOVA	Jungle Sample		Urban Sample	
SOI	df(1,216)	Ayahuasca*n* = 55	Controls*n* = 55	Ayahuasca*n* = 61	Controls*n* = 49	df(1,112)	Ayahuasca*n* = 34	Controls*n* = 43	Ayahuasca*n* = 25	Controls*n* = 14
Transcendent	F = 153.54; p<0.001	5.76 (0.97)	4.03 (1.00)	5.86 (1.03)	3.74 (1.55)	F = 73.68; p<0.001	5.84 (1.05)	4.04 (1.18)	5.87 (0.85)	3.78 (1.46)
Meaning	F = 78.44; p<0.001	5.77 (0.88)	4.88 (0.93)	5.91 (0.74)	4.57 (1.19)	F = 33.88; p<0.001	5.99 (0.63)	4.86 (1.00)	5.76 (0.79)	4.91 (0.84)
Mission	F = 76.62; p<0.001	5.64 (0.90)	4.67 (1.01)	5.55 (0.94)	4.21 (1.33)	F = 38.56; p<0.001	5.46 (1.03)	4.45 (1.00)	5.48 (1.01)	4.42 (1.60)
Sacredness	F = 30.14; p<0.001	5.96 (0.82)	5.10 (0.97)	6.09 (0.79)	4.72 (1.17)	F = 39.83; p<0.001	6.01 (0.80)	5.02 (0.99)	5.96 (0.84)	4.61 (1.18)
Material values	F = 66.78; p<0.001	4.95 (0.84)	4.40 (0.64)	4.82 (0.82)	4.19 (0.85)	F = 21.82; p<0.001	5.30 (0.62)	4.42 (0.54)	4.80 (0.63)	4.18 (0.51)
Altruism	F = 19.32; p<0.001	5.56 (0.86)	5.17 (0.91)	5.36 (0.92)	4.63 (1.10)	F = 5.02; p = 0.027	5.71 (0.90)	5.02 (1.06)	5.35 (0.82)	5.19 (0.76)
Idealism	F = 25.59; p<0.001	4.99 (0.59)	4.62 (0.54)	4.85 (0.60)	4.35 (0.78)	F = 7.33; p = 0.008	4.95 (0.60)	4.57 (0.52)	4.85 (0.69)	4.58 (0.63)
Awareness Tragic	F = 48.64; p<0.001	5.26 (1.01)	4.31 (1.05)	5.25 (1.60)	4.22 (1.07)	F = 16.36; p<0.001	5.20 (1.17)	4.40 (0.91)	5.02 (0.70)	4.20 (1.13)
Fruits of spirituality	F = 91.03; p<0.001	5.96 (0.79)	4.74 (1.21)	6.10 (0.85)	4.20 (1.81)	F = 44.01; p<0.001	5.96 (0.99)	4.81 (1.10)	5.98 (0.93)	4.22 (1.44)
**PLT**	**df(1,216)**	***n*** ** = 52**	***n*** ** = 56**	***n*** ** = 60**	***n*** ** = 52**	**df(1,114)**	***n*** ** = 33**	***n*** ** = 45**	***n*** ** = 25**	***n*** ** = 16**
Score	F = 14.10; p<0.001	114.19 (14.69)	105.84 (16.64)	113.78 (14.61)	104.94 (21.34)	F = 1.78; p = 0.185	116.36 (14.93)	109.80 (24.90)	116.56 (13.80)	113.63 (9.95)
**BIEPS**	**df(1,213)**	***n*** ** = 55**	***n*** ** = 55**	***n*** ** = 55**	***n*** ** = 52**	**df(1,115)**	***n*** ** = 35**	***n*** ** = 48**	***n*** ** = 23**	***n*** ** = 13**
Self-Acceptance	F = 7.46; p = 0.007	8.24 (1.37)	7.95 (1.22)	8.18 (0.96)	7.52 (1.52)	F = 0.50; p = 0.481	8.17 (1.25)	8.23 (1.22)	8.48 (0.89)	8.08 (1.26)
Autonomy	F = 1.89; p = 0.17	7.27 (1.67)	7.29 (1.32)	7.33 (1.47)	6.75 (1.52)	F = 0.85; p = 0.358	7.46 (1.44)	7.02 (1.37)	7.09 (1.24)	7.00 (1.41)
Psychosocial bonds	F = 7.75; p = 0.006	8.33 (1.20)	8.11 (1.10)	8.31 (1.09)	7.54 (1.75)	F = 4.10; p = 0.045	8.57 (1.20)	8.06 (1.34)	8.78 (0.52)	8.31 (1.25)
Projects	F = 6.01; p = 0.015	11.05 (1.17)	10.51 (1.61)	10.87 (1.64)	10.38 (1.73)	F = 1.28; p = 0.260	11.03 (1.60)	10.50 (1.88)	11.17 (1.19)	12.85 (5.81)
BIEPS total	F = 16.17; p<0.001	34.89 (3.29)	33.85 (3.28)	35.44 (4.75)	32.12 (4.44)	F = 6.33; p = 0.013	35.17 (4.13)	33.81 (3.55)	35.48 (2.41)	32.46 (7.88)

The ANOVA column shows results for the main effect of *Group* (ayahuasca users vs. controls). df = degrees of freedom. Aya.  =  ayahuasca-using group. Transcendent-Transcendent dimension; Meaning-Meaning and purpose in life; Mission-Mission in life; Sacredness-Sacredness of life; Awareness Tragic-Awareness of the tragic.

### 5.1. The Spiritual Orientation Inventory (SOI)

In the first assessment, ayahuasca users showed significantly higher scores on all 9 components of the SOI, as revealed by a main *Group* effect on Transcendent Dimension [F(1,216) = 153.54; p<0.001], Meaning and Purpose in Life [F(1,216) = 78.44; p<0.001], Mission in Life [F(1,216) = 76.62; p<0.001], Sacredness of Life [F(1,216) = 30.14; p<0.001], Material Values [F(1,216) = 66.78; p<0.001], Altruism [F(1,216) = 19.32; p<0.001], Idealism [F(1,216) = 25.59; p<0.001], Awareness of the Tragic [F(1,216) = 48.64; p<0.001], and Fruits of Spirituality [F(1,216) = 91.03; p<0.001]. A significant *Group* by *Sample* interaction was found for the latter dimension [F(1,216) = 4.45; p = 0.036], with differences between users and controls being larger in the Urban sample than in the Jungle sample.

In the second assessment, the pattern of results remained unchanged, with higher scores on all components in the ayahuasca-using subjects. Thus, significant *Group* effects were found on Transcendent Dimension [F(1,112) = 73.68; p<0.001], Meaning and Purpose in Life [F(1,112) = 33.88; p<0.001], Mission in Life [F(1,112) = 38.56; p<0.001], Sacredness of Life [F(1,112) = 38.83; p<0.001], Material Values [F(1,112) = 21.82; p<0.001], Altruism [F(1,112) = 5.02; p = 0.027], Idealism [F(1,112) = 7.33; p = 0.008], Awareness of the Tragic [F(1,112) = 16.36; p<0.001], and Fruits of Spirituality [F(1,112) = 44.01; p<0.001].

### 5.2. The Purpose in Life Test (PLT)

Higher scores on this test were found for the ayahuasca-using subjects in the first assessment [F(1,216) = 14.10; p<0.001] but not in the second assessment.

### 5.3. The Psychosocial Well-Being (BIEPS)

The statistical analysis in the first assessment showed significantly higher values for ayahuasca users on the global BIEPS score [F(1,213) = 16.17; p<0.001]. The difference between users and controls was larger in the Urban, as shown by a significant *Group* by *Sample* interaction [F(1,217) = 4.44; p<0.05]. With regard of the individual dimension, users showed higher scores on Self-Acceptance [F(1,213) = 7.46; p = 0.007], Psychosocial Bonds [F(1,213) = 7.75; p = 0.006], and Projects [F(1,213) = 6.01; p = 0.015]. No *Group* effect was found for the Autonomy dimension.

In the second assessment, the *Group* effect on the global score remained [F(1,115) = 6.33; p = 0.013], as did the effect on Psychosocial Bonds [F(1,115) = 4.10; p = 0.045], but no other main effect was found. However, a significant *Group* by *Sample* interaction was found for Projects [F(1,19) = 4.75; p<0.05] revealing lower values for the ayahuasca users as compared to controls in the Urban sample.

## Discussion

In this paper we present data from a field research study in which personality, mental health, life attitudes and neuropsychological performance were assessed in a large number of ritual ayahuasca users and their matched controls.

### 1. Personality

The TCI [49] was used to assess personality. Differences between ayahuasca users and controls were found in several of the temperament dimensions, which are believed to be genetically determined. Higher scores on Reward Dependence (RD) may reflect a feature allowing the group to adapt to a demanding environment such as the tropical rainforest. This interpretation is supported by the significant scores on the RD subdimensions Attachment (RD3) and Dependence (RD4), but not Sentimentality (RD1). This profile is probably useful for life in a small community and in a hostile ecological environment. Participants in the Jungle sample showed a trend to higher scores on Persistence than their urban counterparts. Higher scores on this temperament dimension could explain the adaptation capacity shown by these people to their environment, and the ability to persist as a group despite isolation. Additionally, Harm Avoidance (HA) was lower in the ayahuasca-using subjects, probably reflecting the strength in personality required to undergo regular ayahuasca sessions for long periods of time. It is interesting to note that there were no differences between groups in Novelty Seeking (NS) scores nor in its subscales, including Impulsiveness (NS2). Since high scores in NS and Impulsiveness have been associated with drug use [50], [51], the mere search for new experiences may not be the underlying reason of their involvement with ayahuasca. On the contrary, members of the ayahuasca religions report that the experiences transcend the merely perceptual or recreational aspects of psychoactive drug effects.

The analysis of Character dimensions showed that ayahuasca users scored significantly higher in Self-Transcendence (ST). Since all participants (users and controls) actively practiced some religion, and Character traits can be influenced by personal experience and culture, this finding could be interpreted as a direct effect of ayahuasca use. Self-Directedness (SD), another Character dimension, is consistently lower in the ayahuasca groups, and may also be related to ayahuasca intake. Used in a religious context, the potent psychotropic effects of ayahuasca may strengthen adherence to the doctrine. The lower Self-Directedness (SD) scores found may reflect the greater relevance of the community over the individual. At the same time, there were no differences between users and controls in Cooperativeness (C). So despite greater Self-Transcendence and spirituality in the ayahuasca-using group, willingness to cooperate with others was not different from that seen in more conventional religions. It would be very interesting to assess if subjects who have decided to leave the group and discontinue ayahuasca use share personality traits with the long-term users.

In a group of 15 long term urban ayahuasca users, Grob et al. [28] found lower scores on NS and HA and no differences in RD compared to 15 matched non-users, in line with our own results. The higher RD scores in our study, driven mainly by the Jungle sample, may reflect the difference in environment mentioned above. Another research group has found changes in the Temperament dimensions of the TCI after 6 months of regular ayahuasca use in a religious setting in subjects who were initially naïve to ayahuasca. However, these same subjects did not show changes in the Character dimensions [52]. Based on these findings, a less conservative explanation for the differences observed in Temperament traits in the present study would be that they are a consequence and not the cause of ritual ayahuasca use. This would mean that ayahuasca may induce changes in personality traits traditionally considered inherited. A recent study in which high doses of psilocybin were administered in a supportive setting showed positive long term changes in Openness to Experiences [53]. This temperament trait is considered to be the most substantially heritable trait in the Big Five personality model, and relatively stable through adulthood [54].

### 2. Psychopathology

The analysis of psychopathology indicators showed the important finding that ayahuasca users scored significantly lower on all nine dimensions of the SCL-90-R. The two immediate explanations for this finding are that either ayahuasca has a low potential to induce psychopathology, or that samples of long-term users suffer from a self-selection bias by which only those who do not experience adverse psychological effects continue ayahuasca use. Regarding the second explanation it is worth mentioning that at follow-up lower scores were still seen on most dimensions, despite the loss in sample size. Similar findings have been reported in the literature. In a study where a group of 32 long term US ayahuasca users were assessed with the same instrument, scores were significantly lower than normative data for 7 of the 9 dimensions [31]. Halpern et al. [55] did not find evidence either of psychopathology in a group of peyote (a mescaline-containing cactus) users when compared to controls. Grob et al. [28] did not find evidence of psychopathology in their ayahuasca-taking sample using the CIDI (Composite International Diagnostic Interview), despite the fact that in the retrospective assessment most subjects met criteria for psychiatric disorders prior to their religious use of ayahuasca. Another study with teenage members of an ayahuasca church did not find differences with the control group, but rather showed a tendency to an improvement in some measures of psychopathology [30]. Barbosa et al. [52], [56] also failed to find psychophatological symptoms both in the short-term after a first ritual ayahuasca experience, and at follow up 6 months after continued use. Some participants even showed a decrease in minor psychopathological symptoms.

In summary, though there are case reports describing psychiatric complications following ayahuasca intake [24], [25], it appears that current long-term users do not show higher psychopathology. One study reported that some experienced users even show reduced scores of panic and hopelessness while under the effects of the tea [57]. Future research should assess not only long-term users but also ex-users to evaluate whether adverse psychological effects play any role in the decision to discontinue use. The apparent contradiction between reports of psychiatric crisis after acute ayahuasca and the absence of psychopathology in many chronic users should be studied in more detail.

One last consideration is the potential bias introduced by the self-assessment nature of the SCL-90-R. Subjects may have been inclined to give socially acceptable responses. However, scores on the PST subscale were always higher than 3–4. According to the interpretation norms for the SCL-90-R [58], low scores on this subscale would be indicative of a social desirability bias. Further support for the validity of our present findings is derived from results in the neuropsychological assessment (see below). Psychiatric disorders are commonly accompanied by neuropsychological deficits [59], [60], but these were not observed in the ayahuasca-using subjects in the present study.

### 3. Neuropsychological Functions

Based on the administered tests and the Frontal Systems Behaviour Scales, we found no evidence of neuropsychological impairment in the ayahuasca-using group. Furthermore, in general terms they scored better than their respective comparison groups and these differences were maintained one year later.

These results do not fit the hypothesis of potential frontal impairment secondary to 5-HT_2A_ receptor activation, and are more in line with prior observations in users of psychedelics. Grob et al. [28] found no working memory deficits in their sample of ayahuasca users, but rather an improvement in one memory subset. Da Silveira et al. [29] did not find deficits in the stroop and other neuropsychological tests in their group of adolescent ritual ayahuasca users. These users did not score differently than their control group in most variables. They did fare worse on some memory subtests, but results were within the normalcy range. Halpern et al. [55] did not find neuropsychological impairment in a group of long term peyote users from the Native American Church. Tests included the Stroop test, the Wisconsin Card Sorting Test and working memory tests. Although more research is needed before definite conclusions can be drawn regarding this drug class, based on the available evidence chronic use of psychedelics does not seem to cause cognitive impairment.

The lack of cognitive impairment in our ayahuasca users can not be attributed to a lack of sensitivity of the neuropsychological tests administered, as they were sensitive enough to differentiate between users and non users. The Stroop task and the Wisconsin Card Sorting Test tap various cognitive functions such as selective attention, behavioral inhibition, working memory and goal-directed behavior, and are sensitive to PFC damage [61]. Also, these same tests have been found to detect neuropsychological impairment in various groups of drug abusers. For example, the Wisconsin Card Sorting Test has proven sensitive to detect flexibility impairments in non-addicted cocaine polydrug users (between 1–4 gr. of cocaine per month). This population showed more Perseverative Errors, fewer Categories Completed and worse Conceptual Level Responses than matched controls [62]. Also, the Stroop test was sensitive to detect executive dysfunctions in individuals using alcohol [63], cocaine [64] and amphetamines [65]. The same applies to the Letter-Number Sequencing task [66]. However, the detection of differences between users and non-users is known to be influenced by the length of the abstinence period, the severity and duration of the addiction, the use of multiple drugs and the presence of associated psychopathology [67]. In any case, better performance in the drug-using group is rarely found in the literature other than for the psychedelics. Animal research has shown that 5-HT_2A_ receptor activation plays a role in normal neuropsychological and memory functioning [68]–[71]. Another explanation for the present results has to do with motivation. There is evidence that motivation may in fact improve performance of drug users in neuropsychological tasks [72]. While the recruited ayahuasca users may have been motivated to demonstrate the safety of ayahuasca to researchers, the controls did not obtain any specific benefit from their participation in the study.

Concerning the capacity of the Frontal Systems Behaviour Scales, a self-report questionnaire, to detect impairment, it is worth noting that it has revealed deficits in non-addicted [73] and addicted polydrug users [74], [75]. The lower scores found for our ayahuasca subjects on this measure of prefrontal deficits is consistent with their better neuropsychological performance. This result was found for both samples in the first assessment and in the second.

### 4. Life Attitude and Psychosocial Well-being

All SOI scores were consistently higher in both samples and along time for the ayahuasca users, in consonance with scores on the Self-Transcendence subscale of the TCI. Although a recent study showed no significant differences in spiritually after an ayahuasca session, the magnitude of the observed change was positively correlated with the intensity of the peak of the experience [76]. The qualitative data recorded revealed common spiritual themes among participants [76]. In our first assessment, ayahuasca users showed higher scores on Purpose in Life, although this finding was not replicated one year later. This difference in Purpose of Life may be understood as a consequence of the religious use of ayahuasca, and is compatible with adherence to a religious belief [77]. In line with the above results, ayahuasca users scored higher on subjective psychological well-being. In a previous report where these same participants were assessed on frequency and degree of illicit drug use, ayahuasca users scored lower on the different dimensions of the Addition Severity Index (ASI; [23]). Taken together, the data point at better general mental health and bio-psycho-social adaptation in the ayahuasca-using group compared to the control subjects.

### Limitations

The present study has several limitations. A relevant limitation is that groups were not matched in premorbid IQ, so it is not possible to know whether the differences found in the neuropsychological tests are due to preexisting differences in cognitive abilities or whether ayahuasca used in a ritual context is responsible for the differences observed. Since neuropsychological tasks, and especially working memory tests, are influenced by IQ [78], future studies should control for this variable. From the statistical point of view, the many variables analyzed may have increased the occurrence of type I error. However, given the difficulty of accessing ayahuasca-using populations we chose to administer a comprehensive battery of tests and questionnaires. In addition to the problems associated with self-report questionnaires and the motivational aspects discussed above, a serious limitation, at least in terms of psychopathology, may have been the self-selection bias previously mentioned. Potentially, the assessed individuals may have been those who did not experience any negative neuropsychiatric consequences derived of their continued ayahuasca use. Subjects experiencing adverse consequences may have given up ayahuasca use altogether and may consequently not be among the long-term users accessible to researchers. Future investigation into the neuropsychiatric effects of ayahuasca use should ideally also include people who used ayahuasca regularly in the past but decided to discontinue its use.

### Conclusion

The assessment of the impact of long-term ayahuasca use on mental health from various perspectives (personality, psychopathology, neuropsychology, life attitudes and psychosocial well-being) did not find evidence of pathological alterations in any of the spheres studied. Although ayahuasca-using subjects differed in some personality traits, differences did not fit with a pathological profile. Furthermore, ayahuasca users showed a lower presence of psychopathological symptoms compared to controls. They performed better in neuropsychological tests, scored higher in spirituality and showed better psychosocial adaptation as reflected by some attitudinal traits such as Purpose in Life and Subjective Well-Being. Overall differences with the control group were still observable at follow-up one year later.
